# A mixed-methods feasibility study of a new digital health support package for people after stroke: the *Re*covery-focused *C*ommunity support to *A*void readmissions and improve *P*articipation after *S*troke (ReCAPS) intervention

**DOI:** 10.1186/s40814-022-01197-8

**Published:** 2022-11-19

**Authors:** Janette Cameron, Natasha A. Lannin, Dawn Harris, Nadine E. Andrew, Monique F. Kilkenny, Tara Purvis, Amanda G. Thrift, Tharshanah Thayabaranathan, Fiona Ellery, Garveeta Sookram, Maree Hackett, Ian Kneebone, Avril Drummond, Dominique A. Cadilhac, on behalf of the Recovery-focused Community support to Avoid readmissions and improve Participation after Stroke (ReCAPS) Investigators, Dominique Cadilhac, Natasha Lannin, Helen Dewey, Monique Kilkenny, Nadine Andrew, Jan Cameron, Amanda Thrift, Christopher Levi, Mariko Carey, Geoff Cloud, Rohan Grimley, Sandy Middleton, Vincent Thijs, Toni Aslett, Jonathon Li, Ernest Butler, Henry Ma, Bronwyn Coulton, Kanaga Lagma, Marie Matanas, Rebecca Danton, Natasha Bonanno, Grace Thomas, Naila Pachini, Jennifer Liu, Amanda Thrift, Monique Kilkenny, Jonathan Li, Rebecca Barnden, Amanda Elston, Graeme Hankey, Leonid Churilov, Geoff Donnan, Coralie English, Lana Coleman, Jan Cameron, Verena Schadewaldt, Toni Withiel, Toni Aslett, Eleanor Horton, Brenda Booth, Ida Dempsey

**Affiliations:** 1 Stroke and Ageing Research, Department of Medicine, School of Clinical Sciences at Monash Health, Monash University, Level 3 Hudson Institute Building, 27-31 Wright Street, Clayton, VIC 3168 Australia; 2 Department of Neurosciences, Central Clinical School, Monash University, Melbourne, Australia; 3 Alfred Health, Melbourne, Australia; 4 Department of Medicine, Peninsula Clinical School, Central Clinical School, Monash University, Clayton, Australia; 5 Stroke Division, Florey Institute of Neuroscience and Mental Health, University of Melbourne, Heidelberg, Australia; 6 The George Institute for Global Health, Faculty of Medicine, University of New South Wales, Sydney, Australia; 7 Graduate School of Health, University of Technology Sydney, Ultimo, Australia; 8 Faculty of Medicine and Health Sciences, University of Nottingham, Nottingham, UK

**Keywords:** Stroke, Digital health, eHealth, Feasibility study, Healthcare technology

## Abstract

**Background:**

Evidence for digital health programmes to support people living with stroke is growing. We assessed the feasibility of a protocol and procedures for the *Re*covery-focused *C*ommunity support to *A*void readmissions and improve *P*articipation after *S*troke (ReCAPS) trial.

**Methods:**

We conducted a mixed-method feasibility study. Participants with acute stroke were recruited from three hospitals (Melbourne, Australia). Eligibility: Adults with stroke discharged from hospital to home within 10 days, modified Rankin Score 0–4 and prior use of Short Message System (SMS)/email. While in hospital, recruited participants contributed to structured person-centred goal setting and completed baseline surveys including self-management skills and health-related quality of life. Participants were randomised 7–14 days after discharge via REDCap® (1:1 allocation). Following randomisation, the intervention group received a 12-week programme of personalised electronic support messages (average 66 messages sent by SMS or email) aligned with their goals. The control group received six electronic administrative messages. Feasibility outcomes included the following: number of patients screened and recruited, study retainment, completion of outcome measures and acceptability of the ReCAPS intervention and trial procedures (e.g. participant satisfaction survey, clinician interviews). Protocol fidelity outcomes included number of goals developed (and quality), electronic messages delivered, stop messages received and engagement with messages. We undertook inductive thematic analysis of interview/open-text survey data and descriptive analysis of closed survey questions.

**Results:**

Between November 2018 and October 2019, 312 patients were screened; 37/105 (35%) eligible patients provided consent (mean age 61 years; 32% female); 33 were randomised (17 to intervention). Overall, 29 (88%) participants completed the12-week outcome assessments with 12 (41%) completed assessments in the allocated timeframe and 16 also completing the satisfaction survey (intervention=10). Overall, trial participants felt that the study was worthwhile and most would recommend it to others. Six clinicians participated in one of three focus group interviews; while they reported that the trial and the process of goal setting were acceptable, they raised concerns regarding the additional time required to personalise goals.

**Conclusion:**

The study protocol and procedures were feasible with acceptable retention of participants. Consent and goal personalisation procedures should be centralised for the phase III trial to reduce the burden on hospital clinicians.

**Trial registration:**

Australian New Zealand Clinical Trials Registry, ACTRN12618001468213 (date 31/08/2018); Universal Trial Number: U1111-1206-7237

## Key messages regarding feasibility

- We identify potential benefits, feasibility (inclusive of time-commitment) and acceptability of a new digital health, post-hospital discharge support package (ReCAPS intervention) designed for use after an acute stroke.
- Our training procedures, standard operating manuals goal-setting procedures and use of a REDCap® database accessible via a study iPad supported implementation of our protocol, including consent and baseline data collection, by clinicians within the acute care setting.
- Use of feedback from three complementary sources (participant satisfaction surveys; clinician focus groups; communications with research staff) contributed to the redesign of our ReCAPS package for a definitive randomised controlled trial.

## Background

Stroke is a leading cause of global disease burden [1]. The immediate and long-term consequences of stroke make it challenging for individuals to return to the community and participate in life activities [2]. Adjustments such as learning new behaviours or modifying one’s lifestyle are essential for reducing the risk of future cardiovascular events and supporting recovery, but uptake is poor [3]. The ease with which adjustments occur is multifaceted and relies, in part, on a person’s self-efficacy including beliefs about their capabilities in performing various everyday activities [4]. There is evidence that self-management programmes targeted at self-efficacy improve outcomes such as quality of life and physical function and enhance resilience after stroke [4]. The field of digital health has grown over recent years, in particular with telemedicine services or digital applications [5]. However, it remains unclear whether there is a role for electronic communication delivered in the community for long-term self-management and recovery after a new stroke.

To maximise the effectiveness of self-management strategies and to ensure buy-in from the individual, effort must be made to actively involve the person with stroke in determining goals that are important to them [6]. Goal attainment is also enhanced when support is provided to individuals to achieve their goals [7, 8], but this is difficult outside healthcare settings. Now that use of mobile phones and personal computers is extensive [9], novel digital support programmes that incorporate electronic communication via Short Message Service (SMS) or email may provide the necessary support relating to life after stroke outside healthcare settings [10]. The use of Internet-based programmes [11–13] and electronic communications such as SMS or email [14–17] have gained momentum for addressing secondary prevention of cardiovascular diseases (CVD) in recent years. However, the evidence for digital health programmes that are based on tailored electronic communication to address secondary prevention and recovery after stroke, a disease which tends to occur at older ages than CVD, remains limited.

We aimed to test the feasibility and safety of the *Re*covery-focused *C*ommunity support to *A*void readmissions and improve *P*articipation after *S*troke (ReCAPS) phase II study. The ReCAPS intervention is a tailored digital health self-management post-hospital discharge support package provided for secondary prevention and recovery after stroke. Specifically, this phase II study was initiated to assess the completion of study procedures and feasibility of recruitment and test randomisation processes. We also sought to review and explore the acceptability of the ReCAPS intervention package and options for goal setting, and the potential barriers to recruitment, consent processes and associated training needs as perceived by clinicians working at participating hospitals.

## Methods

### Study design

This was a mixed-methods feasibility trial initiated within three public hospitals (Melbourne, Australia). Feasibility of the trial design and procedures was obtained and summarised from the data collected using screening logs, completion of data report forms and the completion of study procedures. As recommended by Thabane et al. [18], we also obtained qualitative data to supplement the quantitative information. The qualitative component included conducting focus group interviews with clinicians from participating hospitals, communications with research staff and analysis of the open text fields from satisfaction surveys administered to participants on study completion.

### Patient recruitment

Clinicians from stroke units obtained informed consent from eligible patients willing to participate. The inclusion and exclusion criteria are described in Table 1.

**Table 1:** Inclusion and exclusion criteria for the ReCAPS feasibility randomised controlled trial

Inclusion criteria	Exclusion criteria
• Aged ≥ 18 years • Confirmed diagnosis of acute stroke • Discharged directly to a home setting from a stroke unit within 10 days of admission • Have access to the Internet • Self-identify as users of SMS/email technology • Ability to communicate in English • A baseline modified Rankin Score (mRS [19]) of 0–4 • Ability to provide own consent	• Transferred to in-hospital rehabilitation • Significant language impairments that affect the ability to communicate wishes and goals • Poor prognosis (unlikely to survive to 90 days) as assessed by the hospital clinician

*ReCAPS* *Re*covery-focused *C*ommunity support to *A*void readmissions and improve *P*articipation after *S*troke, *SMS* short message service; mRS scoring: 0 = no symptoms; 1 = no significant disability despite some symptoms; 2 = slight disability—unable to carry out all previous activities; 3 = moderate disability, requiring some help to walk; 4 = moderate-severe disability [19]

### Clinician training and other study procedures for the ReCAPS trial

All hospital clinicians and outcome assessors involved in screening, recruitment of patients and outcome assessments completed training sessions with the project manager and trial coordinator. The training included an overview of the study, procedural aspects for screening, recruitment, data capture using a REDCap® [20] (Research Electronic Data Capture) database accessible via a study iPAD and interviewing techniques. To promote the self-selection of health and recovery goals that were meaningful to the participant, all hospital clinicians received training in using the standardised menu-driven goal-setting package from researchers who had developed this [21, 22]. The package supported the setting of person-centred goals according to the SMART (Specific, Measurable, Achievable, Realistic, Timely) criteria [23, 24] to support reliable measurement of attainment using Goal Attainment Scaling (GAS) [23]. As part of the goal-setting training, research clinicians were asked to encourage participants to write down their goals in the educational booklet My Stroke Journey, to share with family and health professionals. The My Stroke Journey booklet is provided at discharge in Australia as part of routine care (https://strokefoundation.org.au/what-we-do/for-health-professionals/hospital-resources/my-stroke-journey).

For each clinician, the goals developed with their first five participants were externally rated for quality using the Smart Goal Evaluation Method (Smart-GEM) [25], by an independent clinical expert. The SMART-GEM scoring system rates each goal according to the extent set goals meet the SMART criteria, with a scoring range of zero (poor quality) to six (good quality) [24]. The SMART-GEM was used as an audit and training tool to ensure standardisation for developing person-centred goals in accordance with all the SMART metrics. If the SMART-GEM score for the participant was less than four, the clinician who developed the goals was given feedback and provided with additional training if required.

Clinical outcomes collected at baseline and 12 weeks post-randomisation included GAS [23]; self-management skills (Health Education Impact Questionnaire (heiQ) [26] and the Stroke Self-Efficacy Questionnaire (SSEQ)) [27]; disability assessed by the modified Rankin Score(mRS) [19]; mood (Hospital Anxiety and Depression Scale (HADS)) [28]; health-related quality of life (EQ-5D-3L) [29]; and longer-term problems affecting physical, social and mental well-being after stroke (Longer-term Unmet Needs Scale (LUNS)) [30]. A study-specific questionnaire on use of healthcare resources and costs was also administered. In preparation for the outcome assessment, the clinical outcome measures were posted to participants 2–3 weeks prior to the due date of their assessment. A blinded outcome assessor contacted participants by telephone 12 weeks post-randomisation, within a window of 14 days. At the end of the outcome assessment, the participant was asked whether they were willing to complete a satisfaction survey, which was either posted or emailed to those who agreed.

The satisfaction survey included open-text and closed questions and had previously been tested [31]. Questions provided the opportunity for participants to share perceived benefits of the programme, willingness to continue with the programme or likelihood of recommending it to other survivors of stroke. Participants in the intervention group were asked additional questions about the type and frequency of electronic messages, appropriateness of message content and perceptions about the adequacy of the length of support in the study.

All clinicians involved in the hospital-based procedures for ReCAPS were invited to participate in a focus group discussion. Focus groups with clinicians were conducted by one researcher who had clinical care and qualitative research expertise. Interviews were conducted face-to-face (x1), via video conference (x1) and via video conference plus telephone (x1). Interviews began with a discussion about the aims of the study and explored views and experiences of the clinicians on the acceptability of the ReCAPS trial protocol, including inclusion criteria, processes for recruitment, outcome assessment and goal-setting. Potential difficulties experienced with implementation or adherence to the protocol and staff training needs were also explored. Discussions were recorded and transcribed verbatim with anonymity of the respondents preserved. Iterative feedback was obtained throughout the trial from the research staff, including trial coordinator, trainers and outcome assessors. Feedback was recorded in study meeting minutes, and adjustments made to process documents were documented.

### Randomisation procedure

**Fig. 1:**
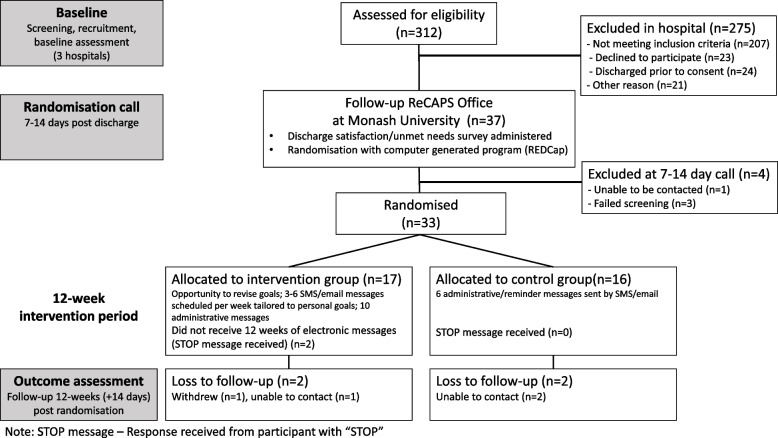
Trial design and CONSORT diagram of participants through each stage of the feasibility study

A randomisation schedule was developed centrally through the REDCap® system by a researcher not involved in the recruitment of participants. The ReCAPS REDCap® database [20] was set to generate the random sequence (1:1 allocation) and stratify by age (<65 or ≥65 years) and level of disability according to the mRS [none-slight (score 0–2), moderate-severe (score 3–4)] [19]. Once the baseline assessment had been completed, participants were contacted between 7 and 14 days from date of hospital discharge by researchers not involved in hospital care or recruitment. At this contact, the researchers reconfirmed the willingness to participate in ReCAPS and performed the randomisation procedures. Allocation was concealed to ensure recruiters, participants, outcome assessors and the study data analyst would be unaware of group allocation (see Fig. 1).

### Recruitment

Recruitment of participants commenced on 28 November 2018 with the final participant recruited on 2 October 2019 (47 weeks). To ensure blinding of participants, the study was described in terms of ‘providing discharge support after hospital’ without explicit details of the differing components of the intervention and control groups.

### Interventions

Both intervention and control groups received the same procedures before randomisation and since this differs from usual care, the control is considered an ‘active’ control. Briefly, the pre-randomisation procedures included the setting of person-centred goals using a 34-item menu to guide the conversation with the participants about choosing between three and five meaningful goals they would like to work on after leaving hospital. All participants were encouraged to select at least one goal related to secondary prevention from the Health goal domain. The Health goal domain covers nine secondary prevention areas such as quitting smoking and controlling blood pressure. To promote a standardised and measurable approach to goal setting, clinicians were encouraged to refer to the manual to help guide them in the process of developing SMART goals with participants. The manual included a summary of best clinical practice recommendations for each goal [32], the goal metrics and worked examples of goals illustrating conformity with the SMART metrics [21, 22]. A summary sheet of the completed goals was then provided to the participant so that these could be shared with family and other health professionals after discharge.

The specific procedures provided to the intervention and control groups following randomisation are outlined in Table 2. The digital health support package is the complete set of components delivered to the intervention group. Following randomisation, we used the iVERVE (Inspiring Virtual Enabled Resources following Vascular Events) platform of >1200 support electronic messages that incorporates a delivery system for tailoring messages to an individual’s goals, as well as delivery of administrative messages [21]. Through the iVERVE platform, the Monash researcher programmed the delivery of electronic messages to commence within 3–4 days of randomisation that continued over 12 weeks. The electronic messages allow two-way communication so that participants could reply to messages, or use “STOP” if they no longer wished to receive them. Both the intervention and active control groups received six electronic administrative messages over the 12 weeks and the intervention group got additional messages specific to supporting achievement of their specified goals.

**Table 2:** Study procedures provided to participants in the intervention and control groups following randomisation

Study procedures	Intervention group	Control group
• Provided with an opportunity to revise, or add more goals, following randomisation (7–14-day call)	Yes	No
• Welcome and administrative electronic messages (*n* = 6) scheduled to be sent from iVERVE during the 12-week intervention, including a message with a link to the Stroke Foundation website sent in the first week.	Yes	Yes
• Between 4 and 7 personalised electronic messages scheduled to be sent from iVERVE weekly, aligned to their goals, their priority and unmet needs	Yes	No
• Additional motivational and secondary prevention messages scheduled during the 12-week intervention	Yes	No
• Ability to respond or send “STOP” messages	Yes	Yes

*iVERVE* Inspiring Virtual Enabled Resources following Vascular Events platform

### Feasibility study outcomes

Assessment of feasibility and the criteria for determining the success of feasibility (in brackets) included:

- Estimating the rates of screening and enrolment by hospital clinicians, determined as the proportion of patients admitted with acute stroke who were screened for study eligibility and enrolled (>30% meeting study eligibility are consented)
- Estimating the rates of retention, measured as the proportion of randomised participants who completed their 12-week outcome assessment within the allocated timeframe (12 weeks post-randomisation + 14 days) (>70% completion of outcome assessment)
- Ascertaining completeness of health measures and goal-setting procedures (>90% completeness)
- Acceptability of the intervention and trial procedures, as measured by a satisfaction survey administered to participants after completion of their final assessment (>80% satisfaction with goal setting processes), and positive feedback from clinician interviews and researcher communications regarding the completion of study processes and time commitment
- Safety, determined by the number of reported serious adverse events (falls or car accidents requiring medical attention, or presentations to hospital) related to the intervention (no serious adverse event related to the intervention).

Intervention fidelity was measured by assessing:

- The number and type of secondary prevention and recovery goals developed by participants (2–5 person-centred goals set by each participant using the goal-setting menu)
- The quality of goals developed by clinicians (SMART-GEM score >4)
- The number of electronic messages successfully sent (no delivery failures)
- The number of ‘Stop’ messages received from participants (<5% request ‘STOP’ to receiving all messages)
- The participants’ engagement with messages (>30% access hyperlinks)

### Sample size rationale

A predefined sample size is not required for a feasibility trial and most will use a pragmatic sample of 12 or more per group, if the sample is representative of the target study [18, 33]. We used a pragmatic sample size of approximately 30 randomised patient participants to evaluate the feasibility of study procedures and protocol.

### Data analysis

Descriptive statistics were reported for the participants’ characteristics, retention and completion of outcome measures by group allocation (intervention or control). Open-ended responses from the satisfaction surveys, and interview transcripts from the focus groups, were analysed using a qualitative content analysis approach [34]; closed questions were summarised descriptively. Methods of constant comparison were used to explore the intervention-context fit and draw together meaning from the data [34].

## Results

### Feasibility of screening, enrolment and retention

A total of 312 patients admitted with acute stroke were screened for study eligibility and 34% (*n* = 105) met the study inclusion criteria (Fig. 1). Consent was provided by 37 (35%) individuals of which 34 (92%) were randomised. There was an observed recruitment rate of 2.8 participants per month per hospital. At the 7–14-day follow-up call, 17 were randomised to the intervention and 16 to control. Reasons for non-eligibility at randomisation (*n* = 4) comprised inability to complete the baseline assessment (*n* = 1), inability to locate the participant (*n* = 1) and a surgical intervention that led to prolonged hospitalisation (*n* = 2). Twenty-nine participants (88%) completed their outcome assessment (intervention *n* = 15; active control *n* = 14). The outcome assessments were completed a median of 104 (range 90 to 154) days from randomisation. One outlier who was difficult to contact had their data collected 154 days post-randomisation. Less than 1% of data were missing at baseline and outcome assessments.

### Characteristics of participants

Participant baseline characteristics are presented in Table 3 with the groups appearing to be well-balanced. Twenty-two participants indicated that email was their preferred method for receiving electronic messages, and all participants indicated a preference for follow-up assessments by telephone call, rather than video-conference call.

**Table 3:** Participant characteristics

Baseline characteristics	Control ***n*** (%) ***N*** = 16	Intervention ***n*** (%) ***N*** = 17
**Demographics**		
Age, mean (SD)	64 (11)	61 (14)
Female	7 (44)	2 (12)
Australian	14 (88)^**d**^	13 (81)
Married/with partner	10 (63)	16 (94)
Live independently	3 (19)	1 (6)
Own home or unit	15 (94)	17 (100)
University educated	5 (31)	3 (18)
**Modified Rankin Scale**		
0-No symptoms at all	1 (6)	3 (18)
1-No significant disability	11 (69)	11 (65)
2-Slight disability	4 (25)	3 (18)
**Self-reported medical history**		
Hypercholesterolaemia	5 (31)	7 (41)
Heart attack	2 (13)	1 (6)
Atrial fibrillation	3 (19)	2 (12)
Hypertension	12 (75)	9 (53)
Sleep apnoea	3 (19)	2 (12)
Respiratory problems	1 (6)	5 (29)
Diabetes	5 (31)	2 (12)
Arthritis	7 (44)	7 (41)
Depression	4 (25)	1 (6)
Anxiety	4 (25)	1 (6)
Cancer	1 (6)	1 (6)
Other serious illnesses	0 (0)	4 (24)
**Lifestyle characteristics**		
Smoking^a^		
Current smoker	3 (19)	1 (6)
Past smoker	5 (31)	7 (41)
Physically active^b^	8 (50)	9 (53)
Alcohol consumption^c^	12 (75)	16 (94)
Healthy eating		
Advised to change diet	10 (63)	6 (35)
>5 servings of vegetables daily	3 (19)	0 (0)
>2 servings of fruit daily	9 (56)	7 (41)

*n* (%) unless otherwise indicated

*SD* standard deviation

^a^Self-report of current smoking status

^b^Undertaking >20 min of vigorous-intensity physical activity > 3 times per week

^c^Self-report consumption of alcohol-containing drinks

^d^1 case missing

### Feasibility—outcome assessment completeness

The 12-week outcome assessments with participants were completed in one telephone session and took between 60 and 90 min. Some participants reported completing mailed copies of the questionnaires (sent to support telephone completion) but did not return them as there was no return envelope. This highlighted the need for study procedures to be revised to enable self-completion as an option. Revised study processes were introduced in April 2019 to facilitate the self-completion of surveys (return by post or electronically). The assessors subsequently reported the remaining assessments taking between 30 and 45 min to complete for those who had completed their surveys prior (electronic or returned paper forms).

Overall, 29 (88%) participants completed the 12-week outcome assessments. However, there were challenges in meeting the outcome assessment timeframe whereby only 12/29 (41%) were conducted in accordance with study protocol (week 12 post-randomisation +14 days). Outcome assessors identified a need to improve participant tracking of follow-up visits to meet protocol requirements. Recommended improvements included setting up reminder notifications in REDCap® to be sent to the outcome assessor and project coordinator for when participants’ assessments were due. The tracking database was improved to record the outcome assessor allocated for the call, the participants’ preferred contact days/times and recording of notes relating to attempted contact calls.

### Feasibility assessment—participant acceptability of the intervention

**Fig. 2:**
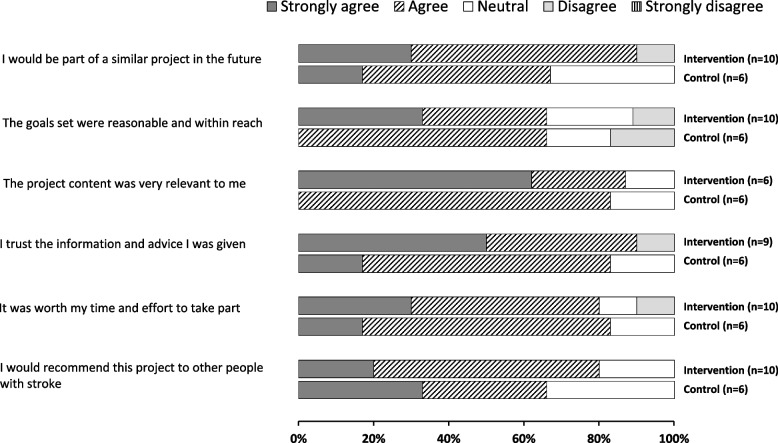
Satisfaction survey feedback from participants (*n* = 16; intervention = 10)

Sixteen participants (48%; 10 intervention: 59%; 6 control: 38%) provided feedback via the satisfaction survey. Most respondents (*n* = 14, >80%) reported that the goal-setting menu was useful to develop person-centred goals, and the clinicians were helpful during this process. Participants were satisfied with the domains categorised on the goal-setting menu and did not provide suggestions for modification. Most participants (*n* = 14, >80%; intervention *n* = 9, control *n* = 8) agreed that it was worthwhile taking part in the study and they would recommend it to others with stroke. Twelve participants responded to the item about the content of the project, and 58% (intervention *n* = 4, control *n* = 4) agreed that they found it relevant to them. Most participants (*n* = 13, >80%; intervention *n* = 8, control *n* = 5) agreed that they trusted the information provided (Fig. 2).

All respondents from the intervention group agreed that they led the process of selecting their own goals. Ninety percent reported that the messages received improved their knowledge of stroke and they were comfortable accessing and responding to the messages (via mobile phone or computer). Most respondents (70%) from the intervention group reported the frequency of the electronic messages received suited them and the 12-week period to receive them was sufficient for their needs. One participant reported a reduced length of time would have been sufficient, and one participant felt the messages should be sent over 24 weeks.

Among intervention participants, most of the respondents (>70%) reported the electronic messages helped to increase self-management, in adopting healthy lifestyle behaviours and in working towards achieving goals. One participant reported they had not received any messages from the ReCAPS team. Five responders (50%) in the intervention group reported not accessing web links for additional information from the messages they received as they did not understand how to access the link, or did not notice the weblinks. All responders in the intervention group reported increased confidence in using electronic devices since being involved in the study.

### Feasibility assessment—clinician feedback

#### Characteristics of clinicians

Six hospital clinicians participated in one of three focus groups (with at least one from each participating hospital): two identified as nurse practitioners, one as a physiotherapist, one as an occupational therapist and two as trial nurses. Clinician participants were all involved in the clinical care of adults after stroke. Most (four of the six) reported prior experience with setting person-centred goals. However, the degree of experience varied between clinicians, with two having held responsibility for leading interdisciplinary goal-setting within stroke rehabilitation teams, while others had only contributed to such teams, or had no prior experience.

#### Hospital clinician feedback on study processes

Clinicians reported several barriers to recruitment, including competing clinical demands, other trials vying for participants and time constraints for completing person-centred goal setting with participants before discharge. Clinicians new to clinical trials highlighted being unsure of how to approach potential participants and provide “the right amount of information” about the trial without “overburdening” them (*Physiotherapist, female, Hospital 2*). Clinicians were responsible for a full clinical caseload in addition to being responsible for study recruitment, limiting their availability to screen and recruit. As highlighted by one clinician: “And I guess what I know is that my role is centrally around acute stroke management so, code stroke calls. This means that there are days where I'm just completely unable to even screen.” (Nurse practitioner, female, Hospital 1).

The inclusion criteria (discharge to home) created a time pressure for recruitment, as discharge destination was not always clear at the time of admission, and the time window for recruitment was often limited by short lengths of stay.

Yeah, it’s, it’s, as I say, it’s always towards discharge, it’s always sort of towards the end of the admission. And it’s typically that I’m providing information and consenting on the same day. (Trial nurse, female, Hospital 1)

It’s a tricky thing with these sort of discharge support type trials, isn’t it because it’s not like a drug trial where you find them on admission, and you’ve got a whole kind of length of time to get through everything you’ve got to. (Occupational Therapist, female, Hospital 2)

Hospital clinicians also reported that baseline measurements were completed over several sessions, due to the time required to complete, and competing clinical demands. Many clinicians left the assessments with the participant for self-completion.

I am always on the lookout for patients who I think are going to be able to be very independent in filling out their questionnaires, because for me being, unfortunately, a slave to the stroke pager means that there are times where I’m just gonna have to go and interrupt our interview... It [the questionnaire] takes quite a long time. And if I can leave it with a patient, great. And usually I'll leave that with them for a few hours. (Nurse practitioner, female, Hospital 1)

Nurses, in particular, reported a lack of confidence in developing person-centred goals while for other health disciplines (physiotherapists or occupational therapists), setting person-centred goals was considered to be consistent with their usual practice. One nurse clinician felt that they had developed new skills during the trial and continued to implement this type of goal setting with all patients at discharge. The tension between spending the required time with the participant for person-centred goal setting whilst meeting health service needs and routine discharge timelines was raised by at least one clinician across each participating hospital, highlighting a universal theme of time constraint in acute hospitalisation.

#### Safety

Five participants (three in the intervention group) reported seven adverse events at the 90-day outcome assessment, all of which were unrelated to the intervention. These included a medication side effect, presentations to the emergency department (*n* = 5) and one planned hospital admission for vascular surgery.

#### Intervention fidelity

Among the 33 participants, a total of 131 person-centred goals were developed across the five major domains. The proportion of participants who selected goals within each of the major goal domains were similar between the intervention and control groups (Table 4). In total, 63 (47%) person-centred goals were developed from the Health domain (e.g. secondary prevention) with the most frequently associated with exercising (*n* = 13), losing weight (*n* = 12) and management of blood pressure (*n* = 10). Overall, return to driving was the goal most frequently selected by participants (*n* = 17, 52%).

**Table 4:** Numbers of participants selecting goals within each major goal domain and according to group allocation

Goal menu items	Overall ***n*** (%) ***N*** = 33	Control group ***n*** (%) ***N*** = 16	Intervention group ***n*** (%) ***N*** = 17
**Major goal domains**			
Your Health	32 (97%)	16 (100)	16 (94)
Mind and body	18 (55%)	9 (56)	9 (54)
Everyday Activities	8 (24%)	4 (25)	4 (24)
Out and About	22 (67%)	11 (69)	11 (65)
Healthcare and Support	5 (15%)	3 (19)	2 (12)

#### Quality assessment of SMART goals developed by hospital clinicians

The person-centred goals developed by hospital clinicians were independently assessed as varying in quality, with SMART-GEM scores ranging from two (poor quality), to six out of six (median score 5.3, Q1 4.6; Q3 5.65). One clinician had quality scores below the SMART-GEM threshold of four; this occurred with four of the eleven goals developed with three participants.

The time taken to set up the messaging schedule for participants in the intervention group took between 50 and 60 min at the start of the trial. This did decrease over time to between 35 and 45 min, with the development of a messaging schedule template according to the number of goals developed.

Participants in the intervention group were sent a total of 1115 messages (average 66 per participant, standard deviation [SD] 10) compared with 101 messages sent to the control group (average 6 per participant, SD 1). There was one occurrence of all scheduled messages failing to be delivered on one day. The issue was rectified within 24 h with the scheduled messages sent. Two participants in the intervention group sent a ‘stop’ message: participant 1 after receiving 41 of 63 messages, finding them “annoying”; participant 2 after receiving 74 of 76 scheduled messages, and who indicated a particular goal had been achieved. Among the 303 messages sent to participants with an embedded bit.ly hyperlink to additional support resources, the hyperlinks were accessed 109 times (36%). The most frequently accessed hyperlink was the Stroke Foundation Enable Me website (44 times, 40%). Eight messages were received from participants in response to questions such as “would you like to speak with someone for further information?”, or “confirm, by reply SMS or email, that you have received our messages?”.

## Discussion

We successfully delivered a post-hospital discharge support package involving person-centred goal setting and electronic messages among 33 individuals. We provide insights into the feasibility, acceptability and fidelity of the study protocol and procedures necessary to refine the study design for a future phase III effectiveness trial. Collecting feedback using the satisfaction survey with participants, focus group interviews with clinicians and meeting minutes from research staff enabled the exploration of the views of all individuals involved in the study. The ReCAPS post-hospital discharge support package was implemented with excellent adherence and <1% missing data suggesting the protocol was well-defined. Qualitative findings provided insights into the acceptability of ReCAPS by exploring the views of clinicians at the three hospitals. Focus groups with hospital clinicians revealed concerns about the time taken to develop person-centred goals prior to discharge, and it is plausible that this tension may have influenced the quality of the goals set. However, all clinicians viewed setting person-centred goals as a key component of the ReCAPS intervention.

Recruitment of participants by hospital clinicians is likely to be supported in a phase III trial, particularly if the length of time to complete the baseline assessment in hospital is reduced. Based on the findings from this feasibility study, it would be expected that with 16 hospitals each recruiting one participant a week, the required sample size of 890 participants for a phase III trial could be obtained within 56 weeks. A feasibility assessment checklist has been implemented to ensure that clinicians who express an interest in collaborating with ReCAPS have the capacity to enrol at least one eligible participant per week and understand the overall recruitment target.

### Major learning points

Synthesising the results identified areas in which study processes could be improved for the implementation of the phase III trial.

#### Hospital clinicians report the recruitment processes to be daunting

Less experienced hospital clinicians valued the training and support provided for recruitment. A peer video featuring advice from a successfully recruiting clinician has been recorded to support multimodal training for future purposes. A study brochure and infographic were developed and implemented to further assist clinicians in explaining the trial to potential participants.

#### Setting person-centred SMART goals takes time and experience

The feasibility evaluation highlighted that setting person-centred goals with participants as part of usual discharge care varied across clinical staff, and participating clinicians reported not always feeling comfortable with the task. This may have been explained by the fact that many of our clinicians were nurses and, unlike allied health professionals, had reported formalised goal-setting processes were not part of their usual practice.

The Trial Executive Committee determined that in the phase III trial, clinicians would use the goal-setting menu to assist participants identify their personal health and recovery goals at baseline. At the 7–14-day post-discharge telephone call, the project coordinator/manager will be responsible for assisting the participant to convert the selected goals into a SMART statement. The person-centred SMART goals developed would continue to be independently audited for quality using the SMART-GEM tool to ensure consistency throughout the trial.

#### Electronic messages were acceptable and relevant

The satisfaction expressed by participants with respect to content, trustworthiness and delivery (frequency and duration) suggests that no changes to the delivery of the ReCAPS support package were required. However, as only half of the intervention participants used the embedded weblinks, the phase III ReCAPS trial includes an explanation to participants at the randomisation call about the potential usefulness of embedded weblinks and how to access them. Participants in the intervention group also receive an electronic message with instructions about using weblinks. In the phase III trial, engagement with electronic messages will be measured by capturing the number of times embedded weblinks are accessed and the number of messages received from participants.

#### Establishing the connection

One participant reported they had not received any messages from the ReCAPS team. Improved administrative processes have been established for the phase III study, so all participants are advised when to expect their first electronic message and reminded to check junk mail if not received. Participants in the intervention group also receive a message asking to confirm the receipt of the first ReCAPS message.

#### Variability in time to collect 90-day outcomes

Improved tracking of outcome assessments due and automated reminder notifications to the outcome assessors via REDCap® have been implemented in the phase III trial. To improve completion of outcome measures and the satisfaction survey, the option for self-completion via REDCap® was established.

This feasibility study has confirmed the importance of the trial as perceived by participants, and the fidelity with study processes and procedures. The high fidelity of the delivery of the protocol led the Investigator group to confidently progress to the phase III trial with refinements introduced using the evidence from this study. Consequently, amendments to the protocol for the transition to a phase III trial were submitted and approved [35]. The phase III trial is a prospective, parallel two-group, double-blind (hospital staff, patients and outcome assessors), multi-site, individual-randomised controlled trial with intention-to-treat analysis. The objective is to test the potential effectiveness, including cost-effectiveness, of the comprehensive electronic self-management support programme provided for 12 weeks after hospital discharge on emergency department presentations or unplanned hospital readmissions and to improve patient self-efficacy over 90 days using a randomised controlled trial design. Feedback from process evaluation will help to understand the acceptability and feasibility of the study intervention and procedures from the perspective of participants, clinicians and researchers who participated in the trial. To ensure the sample size is attained (*n* = 890), it is extended to 13 additional hospitals in Victoria, New South Wales, Queensland, South Australia and Western Australia.

### Study strengths and limitations

A strength of the study was that clinical staff could easily navigate the inclusion criteria and complete 99% of the baseline data. The difference in the number of messages sent between allocated groups demonstrated adherence to the trial protocol. Limitations included the variability in quality of goals set at baseline and challenges with the time required to assist participants to set person-centred goals. Additionally, the number of potentially eligible participants not approached was not well recorded; therefore, the true recruitment proportion remains unclear. Participants were recruited from only three metropolitan hospitals, so it is possible that findings may not be nationally generalisable. A potential limitation was that we did not conduct qualitative interviews with participants as part of the phase II evaluation to determine whether the discharge support package was person-centred. However, the qualitative feedback from development of the goal-setting package and the preliminary testing of the intervention [22, 36] indicated that the goal-setting menu provided an opportunity for the participant to reflect on what was important to them and the health professionals were supportive in guiding the process for developing their goals. Qualitative feedback from participants will be collected in the phase III testing of ReCAPS.

## Conclusions

We successfully demonstrated feasibility of conducting a randomised controlled trial in the acute phase of stroke for participants to receive tailored support for ongoing recovery and secondary prevention post-stroke. Identified barriers to implementing the protocol were modifiable and have resulted in the establishment of a refined protocol. A phase III RCT is feasible and acceptable to clinicians and participants, and a sufficiently powered trial is further required to understand the clinical and cost-effectiveness of the ReCAPS digital health post-hospital discharge support package.

## Data Availability

The datasets used and/or analysed during the current study are available from the corresponding author on reasonable request.

## Abbreviations

EQ-5D-3L: The European Quality of Life questionnaire
GAS: Goal Attainment Scale
HADS: Hospital Anxiety and Depression Scale
heiQ: Health Education Impact Questionnaire
IVERVE: Inspiring Virtual Enabled Resources following Vascular Events
LUNS: Longer-term Unmet Needs Scale
mRS: Modified Rankin Scale
RCT: Randomised Controlled Trial
ReCAPS: Recovery-focused Community support to Avoid readmissions and improve Participation after Stroke
REDCap®: Research Electronic Data Capture
SMART Criteria/Goals: Specific, Measurable, Achievable, Realistic and Time-bound
SMART-GEM: Smart Goal Evaluation Method
SMS: Short message service
SSEQ: Stroke Self-Efficacy Questionnaire
